# Research progress on the application of mixed reality technology in femoral tunnel positioning during anterior cruciate ligament reconstruction

**DOI:** 10.3389/fsurg.2026.1725463

**Published:** 2026-03-13

**Authors:** Binyang Meng, Zi Zhang, Wenhe Li, Qi Wang, Jiangang Cao

**Affiliations:** 1 Department of Sports Injury and Arthroscopy, Tianjin Hospital, Tianjin University, Tianjin, China; 2 Medical School of Tianjin University, Tianjin University, Tianjin, China

**Keywords:** ACL (anterior cruciate ligament), anterior cruciate ligament reconstruction, femoral tunnel localization, mixed reality technology, navigation

## Abstract

Femoral tunnel malposition remains a leading technical contributor to graft failure and revision after anterior cruciate ligament reconstruction (ACLR), particularly in revision surgery, remnant-preserving reconstruction, and multiligament knee reconstruction where arthroscopic landmarks are distorted and tunnel collision must be avoided. Conventional positioning strategies (e.g., clock-face orientation, bony landmarks, and radiographic quadrant methods) provide incomplete three-dimensional control and are susceptible to surgeon dependency and anatomical variability. Mixed reality (MR), distinct from virtual reality (VR) and conventional augmented reality (AR), enables depth-aware holographic visualization, spatial anchoring of patient-specific anatomy, and hands-free interaction, offering a potentially intuitive platform to enhance intraoperative guidance. This narrative review synthesizes evidence on (i) persistent limitations of existing femoral tunnel positioning methods and technology-assisted navigation, (ii) current MR-assisted workflows and intraoperative constraints (registration accuracy and drift, occlusion, sterility and ergonomics), and (iii) transferable lessons from orthopedic MR applications beyond ACLR. Available clinical evidence in ACLR remains limited, but early studies suggest improved tunnel localization consistency without clear short-term functional superiority. Future research should prioritize robust registration/tracking solutions, standardized accuracy endpoints, and well-designed comparative trials to determine whether MR meaningfully improves long-term stability, revision risk, and patient outcomes.

## 1 Introduction

Accurate femoral tunnel placement is a cornerstone of successful ACLR, and non-anatomic femoral tunnel position is repeatedly implicated as a major technical driver of graft failure and the need for revision (1–4). However, achieving reproducible, patient-specific femoral tunnel localization remains challenging because arthroscopic visualization provides limited three-dimensional (3D) spatial information and key landmarks may be obscured or distorted.

These challenges become most pronounced in three scenarios: (1) revision ACLR, where prior tunnels, bone loss, and altered anatomy constrain new tunnel creation; (2) remnant-preserving reconstruction, where footprint visualization is intentionally limited; and (3) multiligament knee reconstruction, where distal femoral bone stock is finite and the risk of tunnel convergence/collision must be actively managed (5–8).

Technology-assisted solutions—including fluoroscopy- or CT/MRI-based navigation and robotic guidance—can improve tunnel placement accuracy in controlled settings (1, 5, 9, 10). Yet their adoption is constrained by workflow complexity, costs, radiation exposure in some protocols, and residual reliance on precise registration and line-of-sight tracking. Moreover, improved radiographic accuracy does not always translate into superior short-term patient-reported outcomes, underscoring the need to define clinically meaningful endpoints and the contexts where advanced guidance offers the greatest value (1, 10, 11).

Mixed reality (MR) has recently emerged as a candidate platform that may address some of these barriers by enabling depth-aware holographic overlay of patient-specific anatomy, intuitive spatial guidance, and hands-free interaction in the operative field. At the same time, MR-guided surgery introduces its own limitations—registration error and drift, occlusion and alignment issues, sterility and ergonomics—which must be critically examined. Therefore, this review is structured around a focused question: Can MR technology overcome persistent limitations of femoral tunnel positioning, and what evidence supports its clinical utility?

## 2 Femoral tunnel positioning in ACLR

### 2.1 Why femoral tunnel accuracy remains difficult?

Femoral tunnel accuracy is fundamentally a 3D targeting problem. Conventional arthroscopic positioning provides a constrained field of view, limited depth cues, and variable visibility of footprint landmarks. As a result, femoral tunnel placement is sensitive to surgeon experience and to patient-specific anatomy. Technical errors are amplified in complex cases where landmarks are altered, soft tissues are intentionally preserved, or multiple tunnels must be created within limited bone stock (5–8, 12).

From a clinical perspective, femoral tunnel malposition is repeatedly cited as a major cause of ACLR failure and revision. Registry data and systematic reviews indicate that revision remains a meaningful burden after both single- and double-bundle reconstruction (13, 14), and multiple analyses identify non-anatomic tunnel positioning as a modifiable risk factor (1–4). These observations support continued efforts to improve reproducible, patient-specific tunnel localization.

### 2.2 Conventional femoral tunnel positioning

This section critically appraises commonly used positioning strategies by the extent of 3D control they provide, their dependence on surgeon interpretation, and their vulnerability in revision, remnant-preserving, and multiligament settings.

Clock-face orientation is intuitive but largely confines guidance to the coronal plane and shows substantial inter-surgeon variability, limiting reproducibility and quantitative evaluation (15, 16). Bony landmark–based approaches (e.g., lateral intercondylar ridge and bifurcate ridge) align with anatomic reconstruction principles but can be unreliable when osteophytes, notch remodeling, or remnant tissue obscures landmarks, and misidentification can shift the tunnel away from the footprint (17, 18).

Remnant-preserving, guide-assisted techniques can facilitate anatomic placement while retaining biologically favorable tissue, but they are case-dependent (acute remnants) and can be difficult for less experienced surgeons; they also raise concerns about impingement and cyclops lesions if exposure and tunnel trajectory are suboptimal (19–21).

Radiographic quadrant frameworks (Bernard quadrant) offer quantifiable coordinates and have become common reference standards in navigation research. However, they reflect population averages and do not inherently account for individual anatomic variation; they also do not guarantee intraoperative 3D reproduction without reliable registration between imaging, arthroscopy, and patient anatomy (10, 11, 22–25). The I.D.E.A.L. concept emphasizes functional graft placement rather than simple footprint centering, but it lacks a universally reproducible intraoperative quantification method, making execution dependent on surgeon judgment and visualization (19, 26). The apex of the deep cartilage (ADC) provides a potentially stable arthroscopic landmark that can be scaled to femoral size, enabling more individualized localization. Nevertheless, ADC identification can be challenging, particularly in cases with cartilage degeneration, altered notch morphology, or limited exposure (23, 27–30) (Table 1).

**Table 1:** Advantages and disadvantages of femoral tunnel positioning theories.

Positioning theories	Advantages	Disadvantages	References
Bernard Quadrant Method (Figure 1)	Gold standard for preoperative planning with navigation systems, accurate positioning, quantifiable tunnel location	Does not account for individual anatomical variations, primarily used in research and theoretical fields	(9, 10, 22–25)
I.D.E.A.L (Figure 2)	Considers ACL anatomy and postoperative outcomes of ACLR	No specific quantifiable method for positioning during surgery	(19, 23, 26, 58, 59)
ADC (Figure 3)	Accurate positioning, quantifiable tunnel location, allows for remnant-preserving reconstruction, adaptable to individual anatomical differences	The apex of the deep cartilage may be difficult to locate accurately	(23, 27–58)

**Figure 1:**
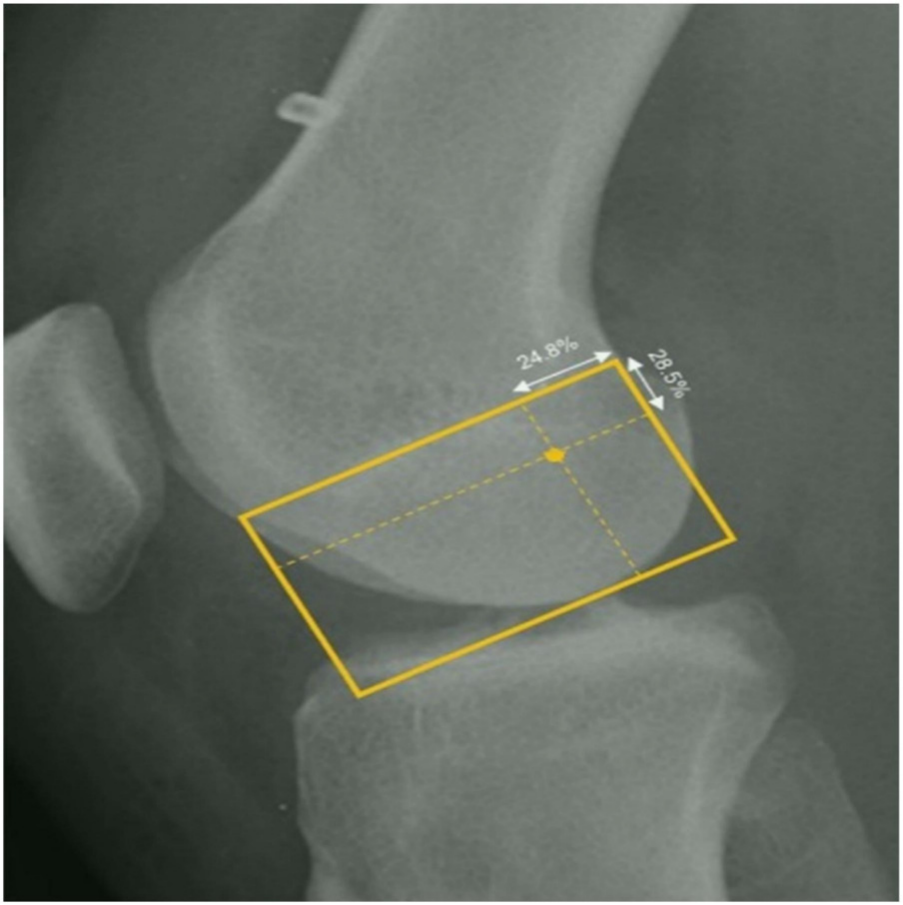
Lateral radiograph of knee with measurement of femoral tunnel positioning using the method by bernard (10).

**Figure 2:**
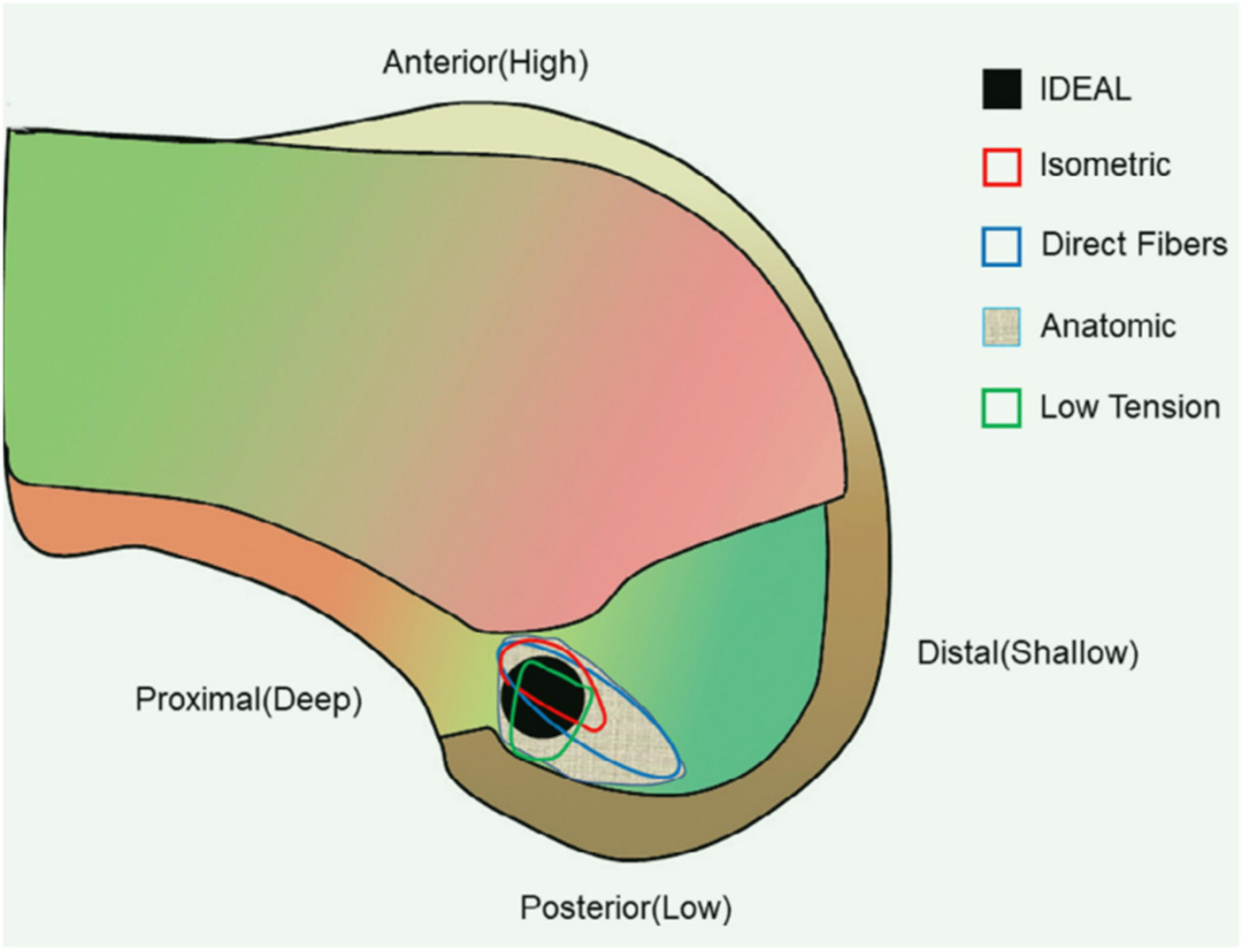
Black circle (I.D.E.A.L. femoral tunnel) locates the ideal placement of femoral tunnel in the single bundle anterior cruciate ligament (ACL) reconstruction (19).

**Figure 3:**
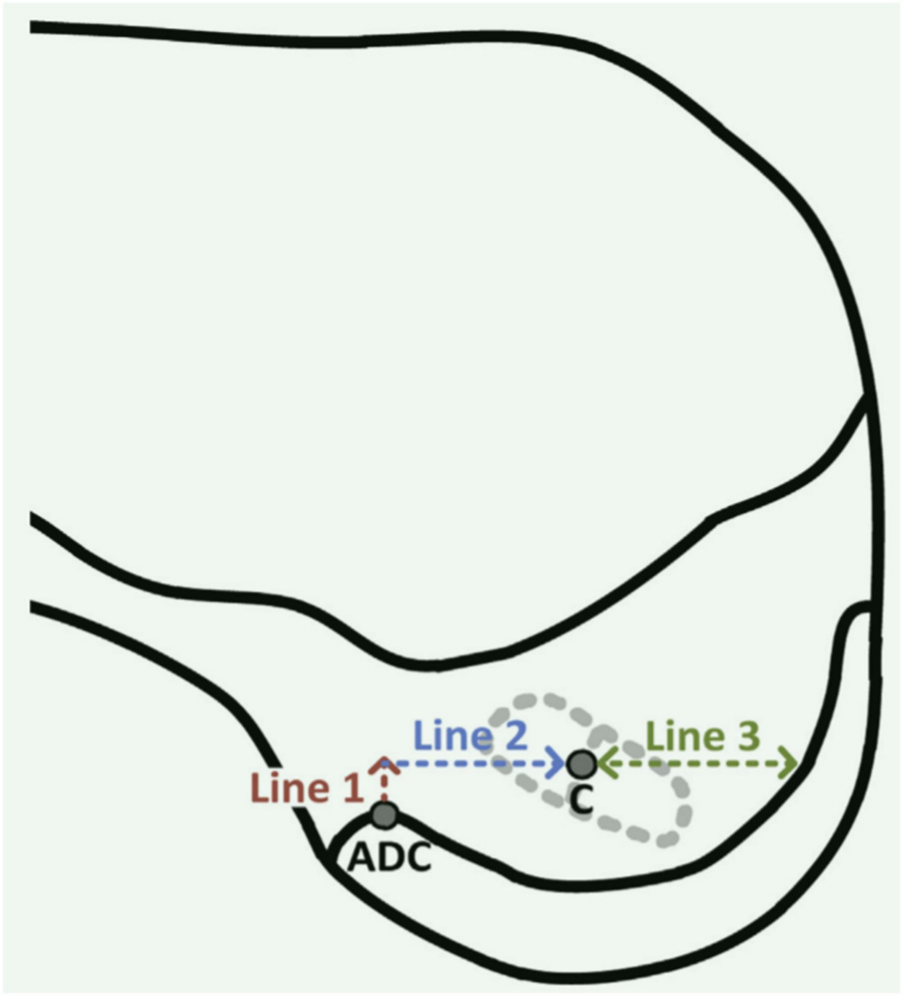
Left femur: mapping the femoral tunnel starting from the apex of the deep cartilage (27).

In revision ACLR, prior tunnels, bone defects, and existing hardware/grafts restrict new tunnel placement and heighten the risk of posterior wall blowout and tunnel convergence. Reliance on subjective arthroscopic landmarks becomes less reliable, and the value of 3D planning and real-time guidance increases (5, 14). In multiligament reconstruction, multiple femoral tunnels must be created within a finite bone envelope, and collision avoidance becomes a primary technical objective. Cadaveric and clinical guideline work emphasizes inter-tunnel relationships, but translating planned trajectories into the operative field remains difficult with arthroscopy alone (6–8).

### 2.2 Limitations of femoral tunnel positioning methods

Currently, the commonly used femoral tunnel positioning methods include the clock-face method, bony landmark method, and anatomical remnant method (17). These methods rely on arthroscopic surgery, where the surgeon uses a handheld guide to position the tunnel based on subjective observation of the ACL attachment site under the arthroscope. However, the surgeon cannot precisely determine the spatial relationship between the bone tunnel and the surrounding bone structure, which can affect the accuracy of the positioning. Since there is individual variability in the ACL attachment points, the positioning process can be challenging. Studies have shown that the accuracy of femoral tunnel placement is influenced by the surgeon's learning curve (9).

Since the introduction of arthroscopic ACLR, the clock-face method has been widely used for femoral tunnel positioning (31–33). Under the arthroscopic view, with the knee flexed at 90°, the apex of the intercondylar notch corresponds to the 12 o'clock position. For the left and right knees, femoral tunnels are drilled at approximately the 1–2 o'clock and 10–11 o'clock positions, respectively, allowing for a simple and effective reconstruction of the ACL. The clock-face method is easy to operate and convenient to apply, offering a quantifiable way to position the femoral tunnel.However, there are significant limitations. First, this method only provides directional positioning of the femoral tunnel in the coronal plane, lacking three-dimensional positioning in the sagittal plane. Second, due to variations in the subjective experience of different surgeons, there is a significant margin of error, making it difficult to achieve consistent results and hindering quantitative evaluation after ACL reconstruction. Given the poor accuracy and precision associated with the clock-face method, it is advised not to use this method in ACL reconstruction (15–17).

The bony landmark positioning method involves placing the femoral tunnel in ACLR at a low and posterior position on the lateral femoral condyle, using the lateral intercondylar ridge and the bifurcate ridge as landmarks for anatomical ACL reconstruction, thereby achieving precise anatomical reconstruction (17, 18). However, this method also has certain limitations. It is not suitable for patients with chronic ACL injuries that result in severe proliferation of the lateral wall of the femoral intercondylar notch or for those undergoing remnant-preserving reconstruction. Additionally, surgeons with limited experience may mistakenly identify the lateral intercondylar ridge as the posterior edge of the lateral femoral condyle, leading to improper femoral tunnel placement and surgical failure.

Both the clock-face method and bony landmark positioning method require the removal of ACL remnants to achieve more precise tunnel positioning. However, remnant-preserving ACL reconstruction has become a growing trend in ACL reconstruction techniques, which increases the difficulty of tunnel placement (12). It was once widely believed that, although ACL remnants possess biomechanical properties (such as proprioception, stability, improved vascularization, and cellular proliferation), they could obscure a clear view of the footprint, making it necessary to remove the remnants to ensure accurate tunnel positioning (34). Recent studies, however, have found that preserving biomechanically compromised ACL remnants during ACLR can lower clinical failure rates and ACL revision surgery rates within one to two years postoperatively, compared to non-remnant-preserving ACLR (35). It has been demonstrated that these ACL remnants retain neuroreceptors and mechanoreceptors, which are crucial for postoperative recovery of proprioception and return to sports (36, 37). The tendon graft-bone interface is a weak link in the early stages following ACLR, with graft failure often occurring in this area (38–40). Regarding graft tendon revascularization, the well-vascularized ACL remnants can accelerate graft “ligamentization” (41, 42). Healing at the femoral tunnel tends to be slower than at the tibial tunnel, partly due to the extensive removal of surrounding remnant soft tissue when exposing the femoral tunnel (5, 43). Remnant-preserving ACL reconstruction can improve graft synovial coverage, promote graft vascularization, enhance proprioception recovery, increase joint stability, block synovial fluid infiltration into the tunnel, and prevent tunnel widening. Although remnant-preserving techniques have shown satisfactory clinical outcomes and provide an effective treatment option for ACL injuries, they require a high level of surgical skill. Postoperative complications such as intercondylar notch impingement and cyclops lesion may occur, which is why remnant-preserving ACLR is only recommended for experienced knee arthroscopic surgeons (19–21).

The remnant-preserving femoral tunnel positioning method with the assistance of a guide is an effective technique for ACLR in cases where remnant-preserving surgery is desired. In acute ACL injuries, when ACL reconstruction is performed early, remnants are often present on the femoral side. The remnant-preserving method with guide assistance involves placing an offset guide at the posterior edge of the lateral femoral condyle, positioning the center of the tunnel at the center of the remnant. This technique allows for anatomical reconstruction while preserving the femoral remnants. Due to its simplicity and accuracy, this method is widely used in clinical practice. However, it is primarily suitable for patients where ACL remnants are still present (17).

The femoral tunnel positioning methods mentioned above also have significant limitations when applied to tunnel revision surgeries and multi-ligament reconstruction following knee dislocations. The revision rates for double-bundle and single-bundle ACL reconstruction are 2.0% and 3.2%, respectively (13). Revision surgeries are costly, and the clinical outcomes of revision ACL reconstruction are generally worse than those of primary reconstruction (1, 14). In ACL revision surgeries, surgeons face several challenges, including dealing with bone defects, misalignment of the original tunnel, managing existing grafts, and providing sufficient space for creating new tunnels (5).

Knee dislocation is a rare but devastating injury that typically involves the rupture of multiple knee ligaments, often necessitating multiple simultaneous ligament reconstructions. This requires the creation of several tunnels in the distal femur. Due to the limited bone stock in the distal femur, there is an increased risk of tunnel convergence and collision. Therefore, the most critical challenge in multi-ligament reconstruction for knee dislocations is how to create multiple necessary tunnels more quickly and accurately while avoiding tunnel collisions (6–8). It has been demonstrated that intraoperative navigation systems can enhance the likelihood of creating optimal tunnel positions and avoiding these pre-existing issues in both revision surgeries and multi-ligament reconstructions (5).

In summary, the success of ACLR largely depends on the precision of femoral tunnel positioning and the surgeon's deep understanding of the anatomy of the ACL femoral footprint. The current primary positioning methods include the bony landmark positioning method and the remnant-preserving method with guide assistance, both based on the concept of anatomical reconstruction. However, considering the limitations of traditional methods such as the clock-face method, bony landmark positioning, and guide-assisted remnant positioning, there is still room for improvement in femoral tunnel positioning techniques for ACLR. Research into precise, personalized, and intelligent intraoperative navigation systems will be the focus of future exploration. Therefore, the development and application of new technologies, such as mixed reality, to enhance surgical precision and efficiency, reduce the risk of complications, and accelerate patient recovery, have become key research directions in the current medical field.

## 3 Mixed reality technology-assisted intraoperative navigation in ACLR

### 3.1 Application of intraoperative navigation systems in ACLR

**Figure 4:**
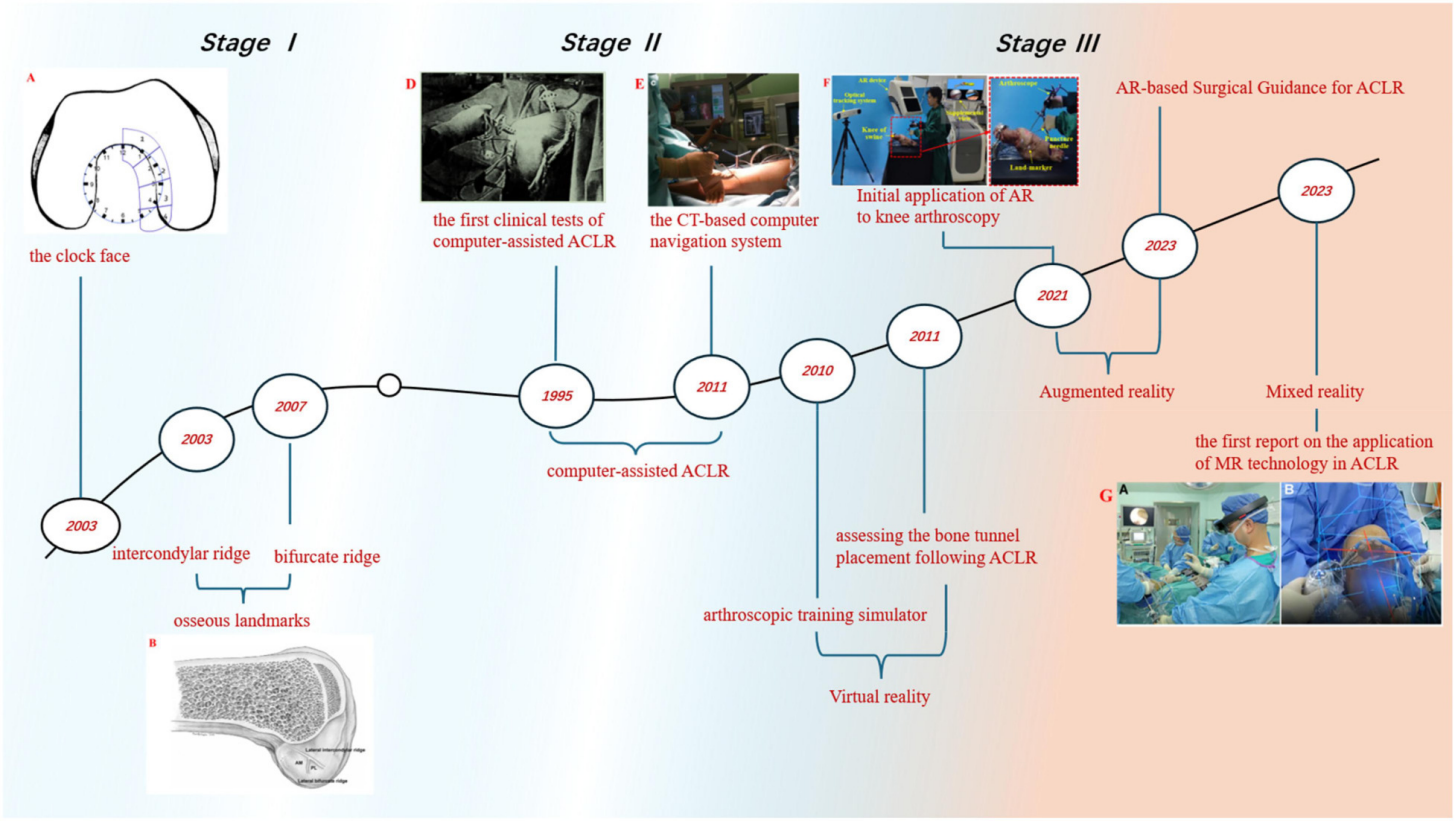
Timeline of ACL navigation technology evolution.

Optical navigation and robotic positioning systems use preoperative CT/MRI-based 3D models, intraoperative registration, and tracked instruments to provide real-time feedback on tunnel trajectory (Figure 4). Multiple studies report improved anatomic tunnel placement compared with conventional arthroscopy (5, 9, 10, 44–46). However, these systems can increase operative complexity and cost, require additional imaging (and sometimes radiation), and remain sensitive to registration errors and line-of-sight limitations. Meta-analyses suggest that although tunnel placement accuracy may improve, short-term clinical outcome scores often do not differ significantly, particularly when procedures are performed by experienced surgeons (1, 10, 11). Accordingly, the strongest value proposition may lie in complex cases and in supporting less experienced surgeons during the learning curve (1, 5).

### 3.2 Mixed reality technology-assisted intraoperative navigation

Virtual reality (VR) replaces the physical environment with a fully virtual scene. Augmented reality (AR) overlays digital information onto the real world, often without robust depth-aware interaction. Mixed reality (MR) is commonly defined as an advanced form of augmentation in which virtual objects are spatially anchored and can be visualized with depth and perspective and interacted with in real time (47–52). In practice, the key promise of MR for surgery is not merely overlay, but controllable, depth-aware, patient-specific 3D visualization aligned to the operative field.

A typical MR-assisted workflow includes: (i) preoperative imaging (CT/MRI) and 3D reconstruction of patient anatomy; (ii) preoperative planning of target femoral footprint coordinates and tunnel trajectory; (iii) intraoperative registration between the virtual model and the patient's anatomy (surface points, bone landmarks, or fiducials); (iv) real-time visualization of the planned tunnel in the surgeon's view via head-mounted display; and (v) hands-free interaction (voice/gesture) to adjust visualization during drilling (24, 53, 54). In current MR-assisted ACLR systems, precise registration and tracking are typically achieved through a hybrid architecture combining external navigation hardware with MR visualization (Table 2).

**Table 2:** Auxiliary registration and tracking components in MR-assisted ACLR.

Component	Function
Optical tracking camera	Real-time tracking of femur and instruments
Fiducial markers/rigid bodies	Provide reference points for 6-DOF pose estimation
Navigation workstation	Data processing and coordinate transformation
Microsoft HoloLens 2	Visualization of holographic model and hands-free interaction
Calibration module	Alignment of tracker, patient anatomy, and MR coordinate system

**Figure 5:**
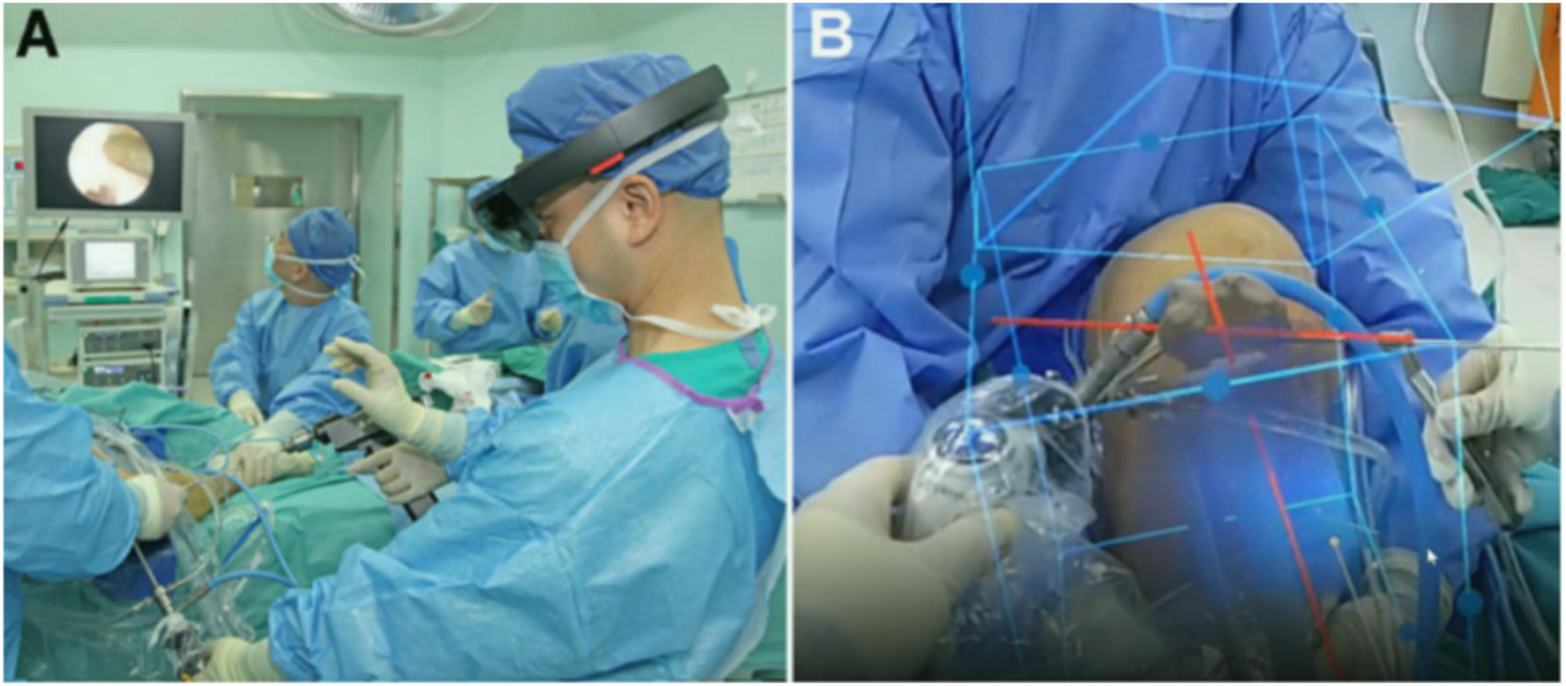
**(A)** the registration process of the surgeon, who wore microsoft holoLens 2 mixed reality holographic display glasses. **(B)** The projected holographic model and surgical site of the registration completed during the drilling of the femoral tunnel (24).

Clinical evidence for MR in ACLR remains limited. In the reported comparative study by Wang et al. (24), MR-assisted single-bundle ACLR demonstrated more concentrated femoral tunnel entry/exit distributions and closer approximation to the preoperative plan, while short-term functional scores improved in both MR and conventional groups without a clear between-group difference at early follow-up (Figure 5). These findings are consistent with the broader navigation literature, where geometric accuracy gains may precede demonstrable differences in patient-reported outcomes (1, 10). At present, clinical evidence specific to MR-assisted ACLR remains limited to a small number of comparative and cadaveric studies, and no large randomized trials are available. Therefore, available evidence is discussed narratively rather than summarized in a standalone ACLR-specific evidence table.

Outside ACLR, MR-assisted navigation has been explored in shoulder arthroplasty, spine instrumentation, and hip arthroplasty (Table 3). Across these settings, MR is primarily used to enhance anatomic visualization, support preoperative planning transfer, and guide instrument trajectories. While reported benefits include improved component positioning or screw placement accuracy and potential reductions in operative time or blood loss in some studies, the generalisability to ACL femoral tunnel drilling depends on solving knee-specific registration and occlusion challenges.

**Table 3:** Applications of mixed reality technology-assisted intraoperative navigation in orthopedic surgeries.

Reference	Surgery	Advantages
Gregory (60)	Reverse shoulder arthroplasty	Accurate anatomical visualization, improved surgical safety, proper prosthesis placement, no postoperative complications
Jennewine (61)	Shoulder arthroplasty	Visualization of complex glenoid deformities, increased preoperative planning accuracy, reduced component misalignment
Wu (62)	Complicated cervical cractures	Accurate anatomical visualization, overcomes limitations of CT 3D reconstruction in depicting nerves and vessels near fractures
Gu (63)	Lumbar pedicle-screw placement	Improved screw placement accuracy, reduced bleeding and surgery time, improved postoperative pain scores
Lei (64)	Total hip arthroplasty	Accurate anatomical visualization, significantly increased surgical accuracy, accelerated postoperative recovery

## 4 Discussion

Current research has demonstrated that mixed reality (MR)-assisted intraoperative navigation systems for ACLR provide numerous benefits in femoral tunnel positioning, including real-time feedback, enhanced precision, and improved visualization. However, the system is still in its early stages of development. In some orthopedic MR applications, there is confusion between the terms “AR” (Augmented Reality) and “MR” (Mixed Reality), although these two technologies are clearly different. MR is defined as a technology that, unlike AR, allows users to visualize the depth and perspective of virtual models, making it a highly valuable tool for anatomical visualization, especially for orthopedic surgeons (52). The fundamental distinction between MR and other technologies is that MR bridges the virtual world and the real world, providing users with a medium that connects both, enhancing the sense of immersion and the integration of reality and virtual environments (47). Therefore, in conducting research on MR-assisted ACLR intraoperative navigation systems, it is crucial to clearly define MR and understand its specific characteristics to fully explore its potential.

Previous studies have shown that intraoperative navigation systems in ACLR may not result in significant differences in postoperative clinical outcome scores compared to traditional ACLR. This has led some researchers to conclude that the use of navigation systems does not necessarily lead to better clinical outcomes, but rather brings increased attention to issues such as the learning curve, high costs, and longer procedure times. In fact, traditional arthroscopic ACLR has been a highly successful clinical intervention since its inception, with a very high patient satisfaction rate postoperatively. Therefore, it is difficult for navigation technology to make a significant improvement in clinical outcomes (5, 11, 55–57). Clinical research should focus more on the use of intraoperative navigation systems in ACLR for precise anatomical positioning, remnant-preserving reconstruction, revision reconstruction (to reduce revision rates), and predicting bone tunnel positions in multi-ligament reconstructions. MR technology-assisted ACL reconstruction allows for accurate femoral tunnel positioning during surgery, improving tunnel reconstruction accuracy without significantly increasing operative time, making it a feasible option for personalized ACLR.

The optimal placement of the ACL tunnel remains a topic of debate, as tunnel positioning should account for individual differences rather than striving for standardization. For experienced surgeons, femoral tunnel positioning in ACLR is already highly accurate. However, for less experienced surgeons, the use of mixed reality (MR) technology-assisted intraoperative navigation systems in ACLR can greatly improve the accuracy of femoral tunnel positioning during early surgeries. Additionally, it can boost their confidence in performing the procedure, helping to shorten the learning curve.

A successful navigation-assisted surgery requires close collaboration across multiple departments. Regardless of the technology used, the surgeon ultimately bears responsibility for the patient, and it is essential to recognize that a thorough understanding of anatomical landmarks is crucial. Navigation is merely a supplementary tool that provides three-dimensional visualization of the patient's anatomy, helps guide preoperative planning, and offers real-time feedback during surgery. If the navigation system malfunctions, the surgery should be able to continue within the standards of conventional care (57). In practical clinical application, the surgeon must always take the lead, with the equipment serving as a support tool. Technology should enhance, not dominate, the procedure, and surgeons should not become overly reliant on the navigation system.

Despite its promise, MR-assisted guidance faces substantial intraoperative challenges. Registration error and drift can accumulate with limb motion, soft-tissue manipulation, and changes in viewing angle. Occlusion and line-of-sight constraints can disrupt tracking and degrade overlay fidelity. Sterility requirements and ergonomics (headset comfort, surgeon fatigue, communication) can influence adoption. Finally, accuracy improvements must be evaluated against clinically meaningful endpoints rather than imaging metrics alone (1, 5, 57).

Key research priorities include: (i) automatic or markerless registration methods with quantifiable error bounds; (ii) hybrid systems combining MR with optical navigation to improve tracking robustness; (iii) standardized accuracy endpoints (e.g., 3D deviation in millimeters/degrees and Bernard coordinates) and reporting; and (iv) trials powered to detect differences in complex-case outcomes, such as revision risk, tunnel collision incidence, graft integration, and long-term stability.

## 5 Conclusion

Persistent limitations of conventional femoral tunnel positioning—particularly incomplete 3D control, surgeon dependency, and vulnerability in revision, remnant-preserving, and multiligament cases—justify continued exploration of advanced intraoperative guidance. MR offers a compelling concept of depth-aware patient-specific visualization and hands-free interaction, but its clinical evidence in ACLR remains early and its technical constraints (registration drift, occlusion, sterility and workflow integration) require rigorous evaluation. Future studies should focus on robust registration/tracking, standardized accuracy metrics, and clinically meaningful endpoints to determine whether MR can translate geometric precision into improved long-term outcomes.
